# A new thermostable Cu(II) coordination polymer: photocatalytic activity and application values on diabetes

**DOI:** 10.1080/15685551.2021.1921341

**Published:** 2021-05-07

**Authors:** Chen-Lu Jin, Shao-Jun Fang, Li Yu, Zhen-Shan Guo

**Affiliations:** a Department of Endocrinology, Zhuji People’s Hospital of Zhejiang Province, Zhuji, Zhejiang, China; b Department of Hepatobiliary Surgery, Zhuji People’s Hospital of Zhejiang Province, Zhuji, Zhejiang, China

**Keywords:** Cu(ii) compound, mixed ligand, photocatalysis, diabetes

## Abstract

A Cu(II) coordination polymer with the composition of [Cu_2_(L)_2_(4,4ʹ-bipy)_2_]_n_·2 n(ClO_4_) (**1**, HL = 4-methyl-L-phenylalanine and 4,4ʹ-bipy is 4,4ʹ-bipyridine), was successfully obtained by the reaction of the mixed ligand of HL and 4,4ʹ-bipy with Cu(ClO_4_)_2_ · 6H_2_O under solvothermal condition. The as-synthesized compound not only has high thermal stability until 275°C but also excellent photocatalytic activity for the methyl blue solution degradation under the irradiation of ultraviolet light. Furthermore, the compound’s treatment activity on the diabetes was determined and its relevant mechanism was also studied. The cytotoxicity or hemolysis toxicity (HC50) of the synthesized compound was also evaluated in this research.

## Introduction

In 2013, the statistics of China on the incidence of diabetes showed that the incidence of type 2 diabetes in adults was 10.4%, of which about 11.1% for men and 9.6% for women [1]. Around 1986, the incidence of diabetes in our country was around 1.03%. In more than 20 years, the incidence of diabetes in our country has increased nearly 10-fold, and there are now more than 100 million diabetic patients [2]. The incidence of diabetes is related to family genetic tendencies and also closely related to usual eating habits. Due to the improvement of people’s living standards in recent years, the incidence of diabetes has also increased than before [3].

As a new class of functional materials, coordination polymers (CPs) have attracted remarkable attention because of their fascinating architectures, at the same time due to their latent applications as the crystalline materials in the gas storage, luminescence, photocatalysis, as well as molecular recognition areas [4–7]. With recent advances in crystal engineering and coordination chemistry, it has been possible to design and synthesize CPs with desired structures and properties via choosing suitable metal ions and organic ligands [8–10]. During the past few decades, the combination of aromatic carboxylate and N-donor ligands has been confirmed as one of the most useful combinations to establish the target CPs, and this building method has also been successfully developed into a dual-ligand synthetic strategy [11–16].

With the development of economy, the discharge of organic dyes has caused serious environment pollution, thus affecting human health. To address the above problem, it is necessary to develop an effective photocatalyst to degrade these stable organic pollutants exploiting the UV/visible/UV-visible light. Recently, lots of Cu(II)/Co(II)/Cd(II)-based CPs have been reported, reflecting outstanding photocatalytic effects for a variety of organic dyes degradation under the irradiation of ultraviolet/visible/ultraviolet-visible light [17–19]. In 2020, Geir Bjørklund proved that zinc and copper have excellent application values in insulin resistance and diabetes mellitus [20]. For the purpose of developing new CPs-based photocatalysts, in this work, we adopted a dual-ligand strategy using 4,4ʹ-bipyridine and the 4-methyl-L-phenylalanine as the organic building blocks to react with Cu(ClO_4_)_2_ · 6H_2_O under solvothermal conditions. Successfully, we obtained a new thermostable Cu(II) compound, and its chemical formula is [Cu_2_(L)_2_(4,4ʹ-bipy)_2_]_n_·2 n(ClO_4_) (**1**, HL = 4-methyl-L-phenylalanine and 4,4ʹ-bipy is 4,4ʹ-bipyridine). The results of X-ray diffraction indicated that the **1** exhibits a three-dimensional cationic skeleton balanced by the lattice perchlorate anions that are filled in the 1D channels. The obtained compound exhibits outstanding photocatalytic effect for the MB solution degradation under ultraviolet light. After serial biological experiments, the application values of the novel compound on the diabetes were detected and the mechanism was explored as well. The blood glucose levels in the body after the compound treatment were measured with blood glucose meter. Besides, the VDAC1 relative expression levels in β-cells was measured through exploiting real-time RT-PCR assay. The results of the cytotoxicity or hemolysis toxicity (HC50) of the synthesized compound indicated that the compound showed no toxicity during disease treatment.

## Experimental

### Materials and instrumentation

The solvents and reagents on the market are of analytical grade and do not require further processing. Through utilizing the analyzer of elemental Vario EL III, the hydrogen, nitrogen, and carbon elements elements were analyzed. The PXRD could be analyzed, and then the data could be collected with the powder diffractometer of PANalytical X’Pert Pro utilizing the Cu/Kα radiation (with λ of 1.54056 Å) with 0.05° step size. The ultraviolet/visible spectrophotometer of Perkin-Elmer Lambda 900 was applied for the measurement of the diffuse reflectance spectra of samples at ambient temperature with the BaSO_4_ plate as standard (with the reflectance of 100%). For the compound **1**, its thermogravimetric analysis (TGA) was implemented via exploiting the thermoanalyzer of NETSCHZ STA-449C under the atmosphere of air with 10°C/min heating rate between 30 and 800°C. And the compound **1**’s fluorescence spectra data was harvested with the Edinburgh Analytical instrument FLS920.

### Synthesis of compound [Cu_2_(L)_2_(4,4ʹ-bipy)_2_]_n_·2 n(ClO_4_) (1)

The mixture synthesized from 0.018 g and 0.05 mmol of Cu(ClO_4_)_2_ · 6H_2_O, 0.1 mmol and 0.018 g of HL, 0.008 g and 0.05 mmol of 4,4ʹ-bipy, 4 mL of CH_3_OH and 8 mL of H_2_O was kept in the Parr stainless steel container with Teflon lining (20 mL); this product acquired was stirred for half an hour, and after that, it was heated for 48 h at 135°C. The complex **1**’s blue massive crystals were separated after gradually cooling the mixture to ambient temperature at 2°C/min rate with the yield of 36% according to Cu(ClO_4_)_2_ · 6H_2_O. Anal. calcd. for the C_40_H_40_Cl_2_Cu_2_N_6_O_12_ (994.76): N, 8.44%; C, 48.25% and H, 4.02%. Found: N, 8.48; C, 48.31 and H, 4.05%.

### X-ray crystallography

The **1**’s data of single crystal has been harvested by the graphite–monochromated Mo–*Kα* radiation (with *λ* of 0.71073 Å) via employing the diffractometer of Oxford Xcalibu E controlled by computer at T = 293(2) K. Dual direct approach is applied to solve the complex **1**’s structure, and then *SHELXL*-2014 is utilized to refine this structure via *F*^2^ based full-matrix least-squares method [21]. The complex **1**’s data of crystallography are calculated in detailed in Table 1. The chosen bond angles (º) and bond lengths (Å) of the complex **1** are revealed in Table S1.

**Table 1.** The data of crystal for the complex **1.**

	1
Formula	C_40_H_40_Cl_2_Cu_2_N_6_O_12_
Fw	994.76
Crystal system	Orthorhombic
Space group	*P*2_1_2_1_2_1_
*a* (Å)	9.400(3)
*b* (Å)	18.874(6)
*c* (Å)	23.071(7)
*α*(°)	90
*β*(°)	90
*γ*(°)	90
Volume (Å^3^)	4093(2)
*Z*	4
Density (calculated)	1.614
Abs. coeff. (mm^−1^)	1.242
Total reflections	32,614
Unique reflections	9382
Goodness of fit on *F^2^*	1.100
Final *R* indices [*I* > 2sigma(*I*^2^)]	*R* = 0.0674, *wR*_2_ = 0.1696
*R* (all data)	*R* = 0.0841, *wR*_2_ = 0.1931
CCDC	2,068,978

### Photocatalytic experiment

The investigation was implemented in accordance with the formerly reported literature [22]. First of all, the solution of methyl blue (MB) (200 mL, 20 mg/L) was added with 50 mg of the compound **1**, and after that, the suspension solution was stirred for approximately 3 h in the dark to construct the equilibrium of adsorption and desorption. Subsequently, the suspension was irradiated by the ultraviolet light from a 175 W high-pressure mercury lamp. During the given time interval, for the reaction mixture, its aliquots were taken regularly and then they were analyzed under environmental temperature via the ultraviolet-visible spectrophotometer. The same procedure was conducted, and the **1** did not add as the blank control experiment.

### Blood glucose determination

After constructing the diabetes animal model and indicated compound treatment, the blood glucose levels *in vivo* were determined with blood glucose meter. This research was accomplished fully in accordance with instructions with minor change. In short, 50 BALB/c mice used in this present research were purchased from the Experimental Animal Center of Nanjing University, with the laboratory animal certificate number of SCXK 2020-0022. High fat feed was given to the animal to induce the animal model of diabetes, and the treatment was carried out by the compound with 1, 2, and 5 mg/kg concentration. After that, the blood glucose levels *in vivo* were then measured with blood glucose meter.

### Real-time RT-PCR

A real-time RT-PCR assay was performed and the VDAC1 relative expression levels in β-cells was detected totally in the light of instruction with minor modifications. Briefly, the animal was provided a high fat feed to induce the animal model of diabetes, and the treatment was performed with the compound with 1, 2, and 5 mg/kg concentration. And then, the β-cells could be collected, and in β-cells, the overall RNA was separated via using the reagent of TRIZOL. After determining the RNA concentration, the concentration was reverse transcripted into the cDNA. Finally, the VDAC1 relative expression levels in β-cells were detected through utilizing the real-time RT-PCR, and the *gapdh* was applied as an internal control gene. The 2^−ΔΔct^ approach was exploited to conduct the statistical analysis.

### Cytotoxicity assay

The Cell Counting Kit-8 was performed in this research to evaluate the inhibitory activity of the synthetic compound on the β-cells viability. This preformation was conducted totally under the guidance of the instructions with only a little change. In brief, the β-cells in the logical growth phage were collected and seeded into the 96-well cell culture plate at the final destiny of 10^4^ cell per well. After 12 h incubation in an incubator at the condition of 37°C, 5% CO_2_, the compound synthesized in this research was added for treatment at serial different dilutions for 48 h incubation. Next, the cell culture medium was discarded and the fresh medium containing CCK-8 reagent was added for 4 h. Finally, the absorbance of each well was measured at 490 mm. This experiment was repeated at least three times.

### Hemolysis toxicity assay

Furthermore, the hemolysis toxicity of the synthetic compound was also determined. This conduction was carried out strictly under the guidance of the protocols with only a little change. The blood used in the hemolysis experiment was taken from adult male New Zealand white rabbits. All the red blood cells were collected and seeded into the 96-well cell culture plate at the final destiny of 10^4^ cell per well. After 12 h incubation in an incubator at the condition of 37°C, 5% CO_2_, the compound synthesized in this research was added for treatment at serial different dilutions for 48 h incubation. Evaluation of the in vitro hemolysis of the material was conducted by measuring the degree of red blood cell lysis and hemoglobin release caused by the contact between the compound and rabbit red blood cells *in vitro*.

## Results and discussion

### Crystal structure of compound 1

**Figure 1.**
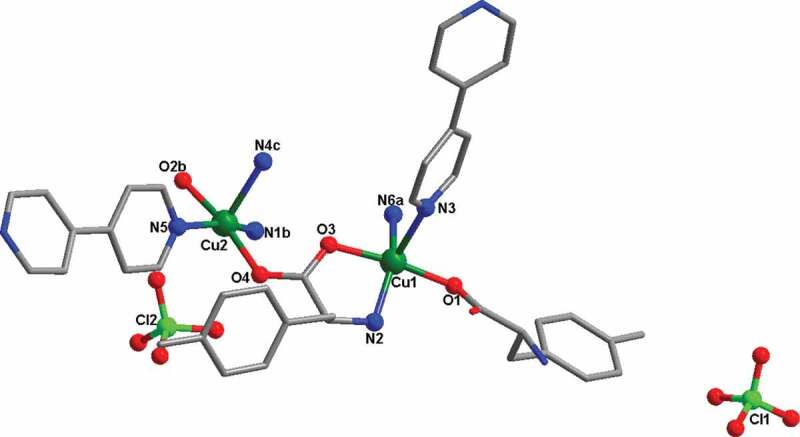
The coordination surrounding view of Cu(II) ions in the complex **1** (symmetry codes: (a) 0.5 + *z*, 1 – *y*, 2.5 – *x*; (b) *z, y*, 1 + *x*; (c) 1 – *z*, 1.5 – *y*, 0.5 + *x*)

**Figure 2.**
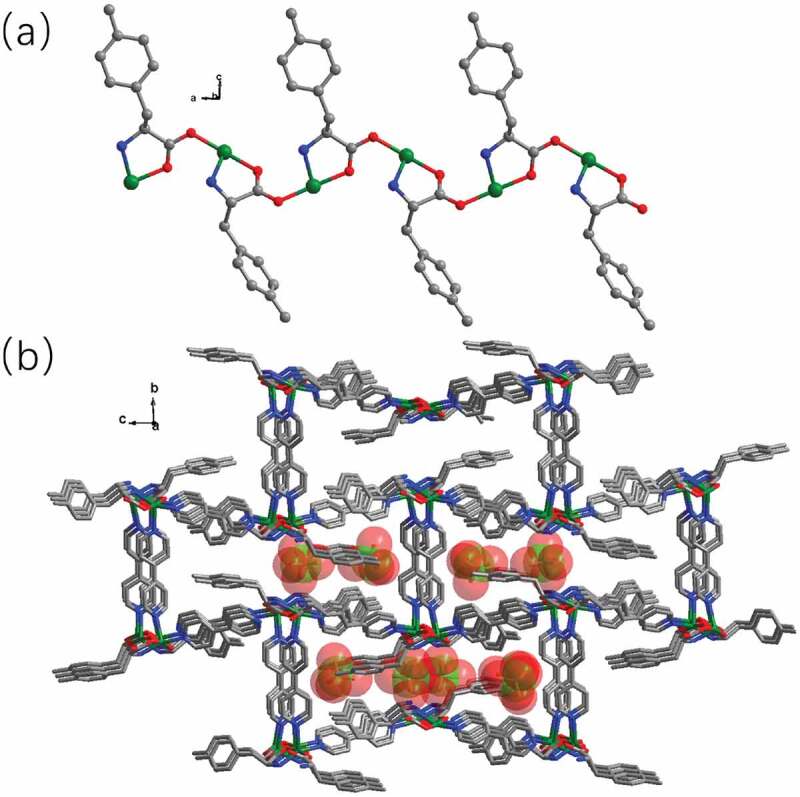
(a) The one-dimensional chain motif established via Cu(II) ions and the ligand of L. (b) The complex **1**’s 3D skeleton with the free perchlorate anions filled in the channels

The analysis of X-ray diffraction suggests that the **1** reflects a 3D cationic framework balanced by the perchlorate anions filled in the channels. As shown in Figure 1, the complex **1**’s asymmetric unit is composed of two ligands of 4,4ʹ-bipy, two ligands of L, two Cu(II) ions, as well as two free perchlorate anions. Both Cu1 and Cu2 are adopted square pyramidal geometries, which were defined through two carboxylic acid O atoms (namely, O3 and O1 for the Cu1 ion, O4 and O2b for Cu2) and one nitrogen donor (N2 for Cu1, N2b for Cu2) from two different L ligands, and other nitrogen donors (N3 and N6a for Cu1, N5 and N4c for Cu2) from two different 4,4ʹ-bipy ligands. Compared with the reported Cu(II) compounds [23], the bond length of Cu–O is between 1.953(5) and 1.980(5) Å, and the spacing of Cu–N bond varies from 2.012(6) to 2.279(6), respectively, which are in the normal range. Each L ligand bridges two different Cu(II) ions in *μ*_2_-(*η*^2^-N, O), O’ mode (Figure S1), and the neighboring Cu(II) ions are connected through the ligands of L into a one-dimensional chain motif (Figure 2a). Next, the 4,4ʹ-bipy ligands further bridge adjacent 1D chains together, affording a 3D cationic framework. In order to balance the charge, the free perchlorate anions filled in the channels of the cationic framework, forming the complex **1**’s ultimate 3D skeleton (Figure 2b).

### Powder X-ray diffraction pattern (PXRD) and TGA

**Figure 3.**
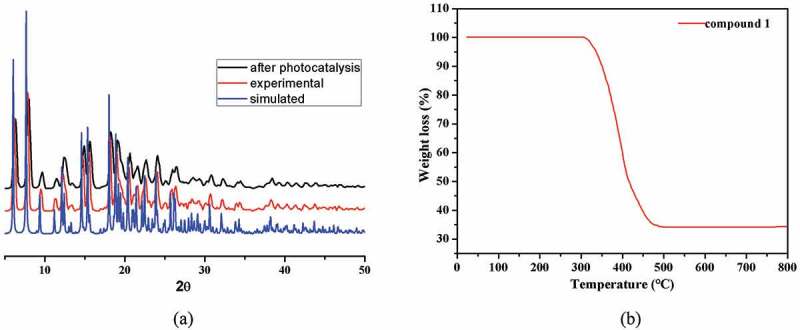
The **1**’s PXRD manners (a); the complex **1**’s curve of TGA (b)

For the sake of checking the as-prepared samples’ phase purity, the complex **1**’s PXRD investigation was carried out under environmental temperature. As displayed in Figure 3a, for the complex **1**, its experimental result is the same as the simulation result of the analysis of the single crystal diffraction, indicating that the as-prepared samples’ purity is high. Furthermore, the stability of **1** in water was also studied *through* the determinations of PXRD, which suggests that compound **1** is insoluble in water. In addition, we also investigated the complex **1**’s thermal stability by the study of TGA under the atmosphere of air from 30°C to 900°C. As illustrated in Figure 3b, when the temperature is less than 275°C, there is no evident weightlessness in the TGA curve, indicating that compound **1** can be stable up to 275°C. From 275°C, significant weight loss occurs that resulted from the organic ligands’ decomposition. The decomposition reaction ends at 533°C, leaving the final residues of 16.71% that may be the copper oxide (calcd: 16.57%).

### Optical property of compound 1

**Figure 4.**
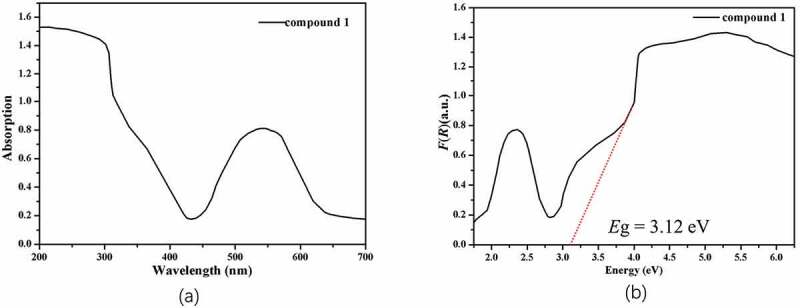
(a) The complex **1**’s ultraviolet-visible diffuse reflectance spectrum. (b) The complex **1**’s Kubelka–Munk transformed diffuse reflectance spectrum

The complex **1**’s ultraviolet-visible diffuse reflectance spectrum was determined under the environmental temperature, and the calculation of its relevant band gap energy (*E*g) was conducted with the equation of Kubelka–Munk, namely, *F* = (1 − *R*)^2^/2 *R*, in which *R* represents the reflectance at specific wavelength. As reflected in Figure 4, between 260 and 430 nm, the compound **1** has a wide absorption band, the absorption peak is about 300 nm, and the **1**’s band gap energy is 3.12 eV, which suggests that compound **1** is an excellent material in the photocatalytic process.

### Photocatalytic property of compound 1

**Figure 5.**
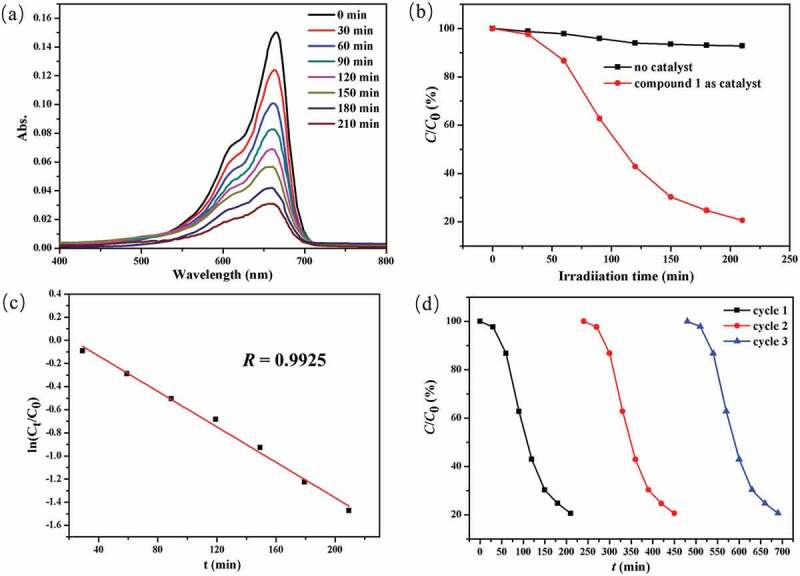
(a) The curve of irradiation time versus MB concentration in the existence of **1**. (b) The photocatalytic decomposition for the solution of MB under the irradiation of ultraviolet light by the complex **1** and the control investigation without utilizing the catalyst. (c) The linear logarithmic plot as the function of time of ultraviolet light irradiation in the existence of **1**. (d) Cycling three runs of the photocatalytic degradation of MB by **1.**

In the work, using the MB as model dye, the complex **1**’s photocatalytic effect under the irradiation of ultraviolet was studied. As revealed in Figure 5a, the characteristic absorption peak of MB at approximately 662 nm slowly reduced with the increase of irradiation time in the presence of **1**. It can be found that the degradation efficiency can reach 79.44% after 210 min irradiation time, which can be calculated by the equation of *η *= (*C*_0_–*C*_t_)/*C*_0_ where *η, C*_0_, and *C*_t_ represent the degradation efficiency, initial concentration of dye solution, and final concentration of dye solution, respectively (Figure 5b). However, the blank investigation revealed that the degradation rate was 7.26% in 210 min without complex **1**. It is clear that compound **1** can be used as an effective catalyst for the photodegradation of MB. Compared with previously reported literatures, compound **1** reported here shows relatively poor activity for degradation of MB solution [24,25]. Notably, the degradation reaction of MB in the presence of **1** follows the first-order kinetic equation ln(*C*_t_/*C*_0_) = −*k*t + *b*, and the degradation rate constant of *k* value is 0.0077 min^−1^ (Figure 5c). Furthermore, the stability test of **1** for degradation of MB was explored by three cycles experiments, and the degradation efficiency remained above 78% after three cycles (Figure 5d). After the third cycle photocatalytic trial, the samples of **1** were separated by centrifugation, and further dried at 100°C for 12 h. Its pattern of PXRD is in accordance with the **1**’s original architecture, exhibiting that the structure of **1** is stable during the process of photocatalytic reaction.

### Compound significantly reduces the blood glucose levels dose dependently

**Figure 6.**
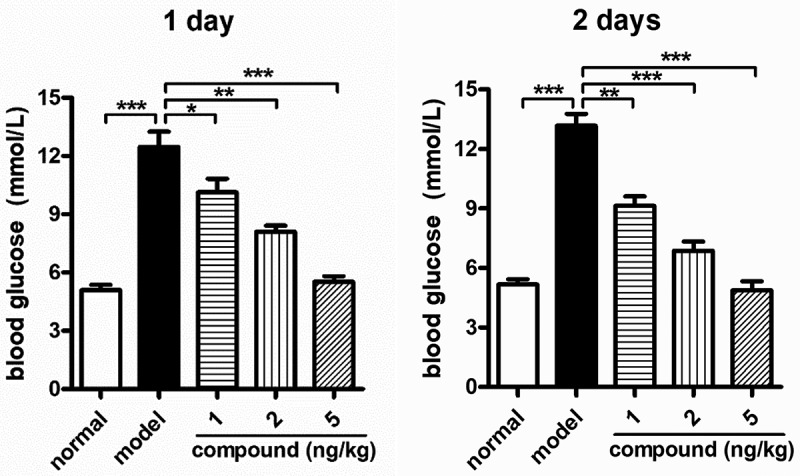
Significant reduction of the blood glucose levels *in vivo* after the compound treatment. The diabetes animal model was constructed and then the treatment was carried out with the compound at 1, 2, and 5 mg/kg concentration. The blood glucose levels *in vivo* were measured with blood glucose meter

After the synthesis of the new compound, its application values on the diabetes were evaluated. So, the blood glucose meter was used to determine the blood glucose levels after the indicated treatment. As the outcomes illustrated in Figure 6, we can observe that the model group has a significantly enhanced blood glucose level in comparison with the control group. However, after treating through the novel complex, the levels of blood glucose were reduced in a dose and dependent manner, suggesting the novel compound’s excellent application values against the diabetes disease.

### Compound obviously downregulated the relative expression levels of the VDAC1 in the β-cells

**Figure 7.**
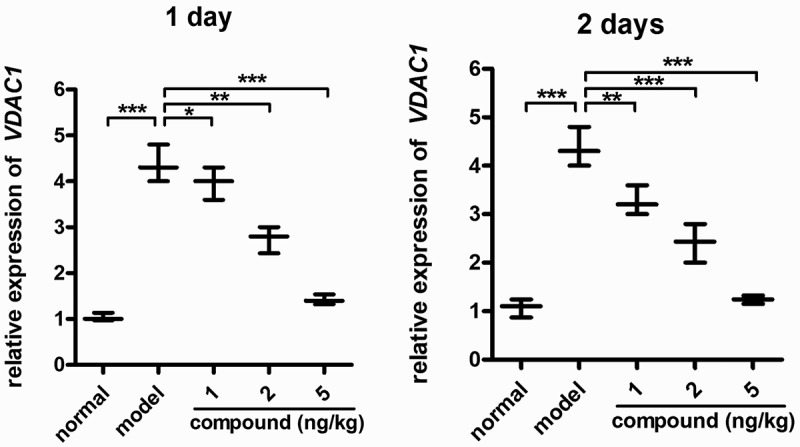
Remarkably downregulated VDAC1 relative expression levels in β-cells. The animal model of diabetes was created and then the treatment was performed with the compound at 1 , 2, and 5 mg/kg concentration. The real-time RT-PCR was implemented, and the VDAC1 relative expression levels in β-cells were accomplished

During diabetes, normal function of β-cell can be restored by blocking the protein VDAC1 expression, and in the previous research, we also confirmed the compound’s inhibitory effect against the levels of blood glucose *in vivo*. As a result, in this work, the real-time RT-PCR was accomplished to determine the expression levels of VDAC1 in β-cells. The outcomes in Figure 7 suggest that in contrast to the model group, this compound could remarkably decrease the VDAC1 relative expression levels in β-cells. This compound’s inhibition revealed the dose- and time-dependent fashion.

### The compound exhibited no cytotoxicity

**Figure 8.**
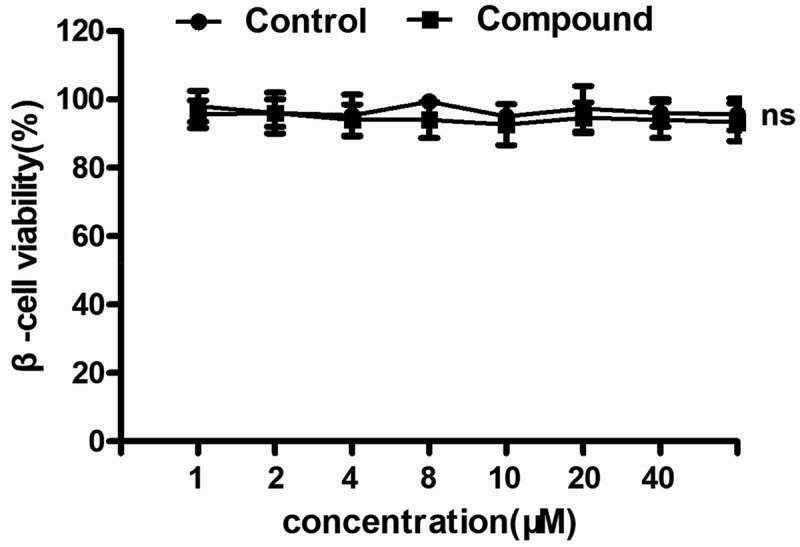
No cytotoxicity of the compound on β-cells. The β-cells in the logical growth phage were collected and treated with serial different dilutions of the compound for 48 h incubation. The viability of the new compound was evaluated with CCK-8 assay

As previously described, the compound exhibited excellent applications on the treatment of diabetes through increasing the function of β-cell function. However, the cytotoxicity of the new compound still needs to be explored. Thus, the CCK-8 assay was conducted in this research, and the inhibitory activity of the new compound was assessed. As the results show in Figure 8, we can see that compared with the normal group, the compound showed no inhibitory effect of the viability of the β-cells. There was no significant difference between these groups.

### The compound exhibited no cytotoxicity

**Figure 9.**
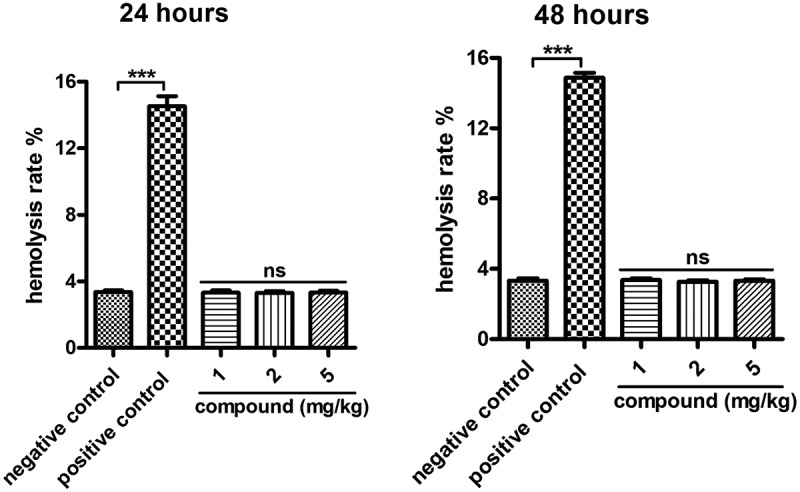
No hemolysis toxicity of the compound on β-cells. Blood used in the hemolysis experiment was taken from adult male New Zealand white rabbits and incubated with serial different dilutions of the new compound for 48 h incubation. The degree of red blood cell lysis and hemoglobin release caused by the compound was evaluated

In addition to the cytotoxicity evaluation, the hemolysis toxicity (HC50) of the synthesized compound was also determined. So, the hemolysis assay was carried out to ensure the application values of the new compound during vein injection. The results in Figure 9 showed that there was a 3.3% hemolysis rate in the control group. While after the treatment of the new compound we can see that the hemolysis rate of the compound to blood is 3.4%, which is less than the standard 5%, which is non-hemolytic reaction.

## Conclusions

To recap briefly, we have acquired a new Cu(II) CP, and its chemical formula is [Cu_2_(L)_2_(4,4ʹ-bipy)_2_]_n_·2 n(ClO_4_), which was created via the solvothermal reactions between Cu(ClO_4_)_2_ · 6H_2_O, 4,4ʹ-bipy and HL. Under the synergistic coordination effects of L and 4,4ʹ-bipy ligands, all Cu(II) ions are linked into a three-dimensional cationic skeleton that was balanced through the lattice perchlorate anions. The complex **1**’s outstanding photocatalytic effect for the MB degradation under the irradiation of ultraviolet makes it possible to be used in the purification of wastewater. The results of the blood glucose analysis showed that the blood glucose could be significantly reduced under the treatment of the new compound. In addition, the compound could also obviously decrease the VDAC1 relative expression levels in β-cells dose dependently. The results of the cytotoxicity or hemolysis toxicity (HC50) of the synthesized compound indicated that the compound showed no toxicity during disease treatment. Ultimately, we obtained the summary that this complex has good protein activity and is an ideal candidate for the treatment of diabetes through increasing the function of β-cell function.

## Supplementary Material

Supplemental Material

## Data Availability

Selected bond lengths (Å) and angles (^°^) for compounds **1** (Table S1), the coordination mode of L ligand (Figure S1), and the information could be found in the supporting information file.
