# Supplementary material for: A new thermostable Cu(II) coordination polymer: photocatalytic activity and application values on diabetes
Source: Des Monomers Polym. 2021 May 7;24(1):136–44. doi: 10.1080/15685551.2021.1921341 (PMC8118421; doi:10.1080/15685551.2021.1921341)
Supplement: Supplemental Material [file TDMP_A_1921341_SM7843.doc]

Table S1 Selected bond lengths (Å) and angles (°) for compounds **1**.

| **Compound 1** |  |  |  |
| --- | --- | --- | --- |
| Cu(1)-O(1) | 1.955(5) | Cu(1)-O(3) | 1.953(5) |
| Cu(1)-N(2) | 2.012(6) | Cu(1)-N(3) | 2.261(7) |
| Cu(1)-N(6) | 2.054(6) | Cu(2)-O(2)^2^ | 1.980(5) |
| Cu(2)-O(4) | 1.973(5) | Cu(2)-N(1)^2^ | 2.025(6) |
| Cu(2)-N(4)^3^ | 2.279(6) | Cu(2)-N(5) | 2.032(6) |
| O(1)-Cu(1)-N(2) | 93.2(2) | O(1)-Cu(1)-N(3) | 93.0(3) |
| O(1)-Cu(1)-N(6)^1^ | 90.9(2) | O(3)-Cu(1)-O(1) | 171.6(2) |
| O(3)-Cu(1)-N(2) | 82.3(2) | O(3)-Cu(1)-N(3) | 95.3(3) |
| O(3)-Cu(1)-N(6)^1^ | 89.9(2) | N(2)-Cu(1)-N(3) | 116.7(3) |
| N(2)-Cu(1)-N(6)^1^ | 152.4(3) | N(6)^1^-Cu(1)-N(3) | 90.3(3) |
| O(2)^2^-Cu(2)-N(1)^2^ | 82.7(2) | O(2)^2^-Cu(2)-N(4)^3^ | 90.6(2) |
| O(2)^2^-Cu(2)-N(5)^2^ | 90.2(2) | O(4)-Cu(2)-O(2)^2^ | 169.9(2) |
| O(4)-Cu(2)-N(1)^2^ | 94.7(2) | O(4)-Cu(2)-N(4)^3^ | 99.5(2) |
| O(4)-Cu(2)-N(5) | 89.3(2) | N(1)^2^-Cu(2)-N(5) | 161.4(3) |
| N(5)-Cu(2)-N(4)^3^ | 93.5(2) | N(1)^2^-Cu(2)-N(4)^3^ | 103.8(3) |

Symmetry codes: (1) 5/2 – *x*, 1 – *y*, 1/2 + *z*; (2) 1 + *x*, *y*, 1 + *z*; (3) 1/2 + *x*, 3/2 – *y*, 1 – *z*.

**
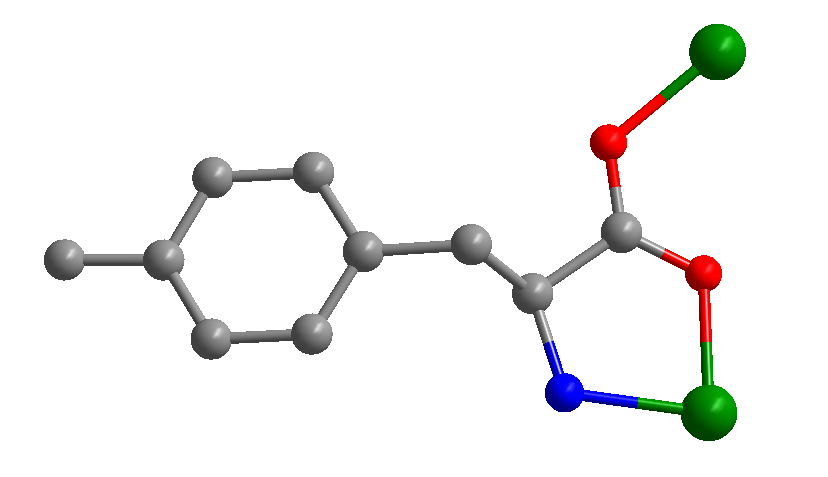
**

**Fig.S1.** The coordination mode of L ligand.
